# Inducing tumor-intrinsic innate immune response to break cancer immunotherapy resistance

**DOI:** 10.3389/fmed.2026.1892906

**Published:** 2026-07-03

**Authors:** Jessica A. Blandino, Takahiko Murayama

**Affiliations:** Department of Cell Biology, SUNY Downstate Health Sciences University, Brooklyn, NY, United States

**Keywords:** cancer therapy, immune checkpoint blockade, nucleic acid sensors, tumor microenvironment, viral mimicry

## Abstract

Immune checkpoint blockade (ICB) therapy targeting the PD-1/PD-L1 axis has dramatically transformed cancer treatment. However, durable responses are limited to a subset of patients, particularly for those with solid tumors. Therefore, understanding the resistance mechanisms and developing a novel therapeutic strategy are urgent priorities. The tumor microenvironment (TME) is a key determinant of ICB therapy responsiveness, and resistance mechanisms are heterogeneous. Recent studies have shown that a major contributor to resistance is an immunologically “cold” TME that contains very few infiltrating immune cells. Inducing tumor-intrinsic innate immune responses through the viral mimicry response, in which cytoplasmic nucleic acid sensors are activated by aberrantly accumulated nucleic acids, represents a promising strategy to convert “cold” tumors to “hot” (immune-inflamed) and enhance ICB efficacy. In this review, we summarize the current understanding of TME-mediated resistance to ICB and introduce therapeutic approaches that trigger viral mimicry responses in cancer cells, including epigenetic therapies, agents that perturb nucleic acid metabolism, and DNA damage inducers, and discuss opportunities for combining viral mimicry-based strategies with ICB therapy to overcome resistance.

## Introduction

Tumor immunotherapy with immune checkpoint blockade (ICB) aims to relieve suppression of anti-tumor immune responses mediated by cancer cells. In general, T cell activation is regulated by a balance between stimulatory and inhibitory ligand-receptor interactions, collectively referred to as immune checkpoints (1). Within the tumor microenvironment (TME), T cells, cancer cells, dendritic cells, and macrophages engage with each other through these molecular interactions (2). Cancer cells are particularly important mediators of immunosuppression owing to their often-upregulated expression of inhibitory ligands (3). Thus, blocking these inhibitory ligand-receptor interactions can restore or induce anti-tumor immune responses.

The successes of the ICB agents ipilimumab (4) and nivolumab (5) against metastatic melanoma in the 2010s marked a major breakthrough in tumor immunotherapy. In addition to ipilimumab, which targets cytotoxic T-lymphocyte antigen-4 (CTLA-4; expressed on T cells), and nivolumab, which targets programmed cell death protein 1 (PD-1; on T cells), there are multiple other approved ICB agents targeting CTLA-4, PD-1, and the PD-1 programmed cell death-ligand 1 (PD-L1; expressed on cancer and other cells) (6–8). More recently, relatlimab (9), which targets lymphocyte-activation gene 3 (LAG-3; on T cells), was approved by the U.S. Food and Drug Administration (FDA) exclusively in combination with nivolumab, and additional checkpoint targets are currently under evaluation in clinical and preclinical studies (10–12).

As clinical investigation has progressed, it has become evident that the cellular and non-cellular components and functional state of the TME are critical in determining the sensitivity of tumors to ICB. “Hot” tumors, characterized by abundant infiltration of anti-tumor immune cells, as frequently observed in melanoma, non-small cell lung cancer, and renal cell carcinoma, tend to respond well to ICB, whereas “cold” tumors with sparse immune infiltrates in the TME typically exhibit poor responses (13–15). Resistance to ICB arises through diverse mechanisms, but many converge on the reduction in the number or activity of effector immune cells in the microenvironment. This is consistent with the mechanism of action of ICB, which depends on effective interactions between immune cells and cancer cells.

In this review, we summarize components of TME and mechanisms of resistance to ICB, describe stratigies to induce tumor-intrinsic innate immunity via viral mimicry, and review clinical approaches that combine viral mimicry with ICB agents to overcome resistance.

## Tumor microenvironment components and determinants of ICB therapy

The TME is a complex, dynamic ecosystem composed of (1) cancer cells themselves; (2) non-malignant cellular components including immune cells (dendritic cells, macrophages, neutrophils, natural killer cells, cytotoxic T cells, memory T cells, regulatory T cells, and B cells) and stromal cells (cancer-associated fibroblasts, endothelial cells, and pericytes); and (3) non-cellular elements such as the extracellular matrix (ECM), soluble mediators (growth factors, cytokines, and chemokines), and metabolites (16–19). Although both cellular and non-cellular components have been reported to contribute to tumor development and therapeutic response (19), a key determinant is the degree and quality of T cell infiltration and activation. Effective anti-tumor immunity by T cells requires coordinated steps: antigen processing and presentation by antigen-presenting cells (APCs) such as dendritic cells, priming naïve T cells in the draining lymph node, activation and expansion of cancer cell-specific T cells, recruitment of those T cells into the TME, and recognition and killing of cancer cells (20). Thus, disruption of any of these steps can lead to primary or acquired resistance to ICB.

In addition to whether a tumor is “hot” or “cold” (13), many other tumor- and TME-specific factors determine responsiveness to ICB. First, the expression level of key biomarkers on cancer cells, such as PD-L1, can be an important determinant (21); indeed, PD-L1 expression is used in companion diagnostics to select patients for PD-1/PD-L1 blockade in many cancer types (22). Mechanisms driving T cell exclusion from “cold” TMEs include dense ECM deposition, aberrant tumor vasculature, and chemokine dysregulation that impairs T cell recruitment (23). The antigenic landscape and antigen presentation capacity also affect ICB outcomes. High tumor mutational burden and elevated neoantigen load can increase the likelihood of immunogenic epitopes (24), but effective neoantigen presentation requires intact major histocompatibility complex class I (MHC I) expression and competent APCs (25, 26). For example, small cell lung cancers frequently exhibit high tumor mutational burden, yet show low ICB responses and possess “cold” TMEs due to loss of MHC expression (15, 27). Tumor-intrinsic loss of antigen presentation machinery (β2-microglobulin loss, HLA downregulation) (28) or APC dysfunction (16) within the TME undermines T cell recognition and permits immune escape. Metabolic changes in the TME, including hypoxia and acidosis, can further dampen T cell function and promote exhaustion programs (29). As discussed further in the next section, infiltration of immunosuppressive cellular components—regulatory T cells (Tregs), myeloid-derived suppressor cells (MDSCs), M2-like tumor-associated macrophages (TAMs), and tolerogenic dendritic cells—actively inhibits effector T cells. Understanding the interplay between tumor-intrinsic programs and the TME is therefore essential for devising effective therapeutic strategies.

## Mechanisms of TME-mediated resistance to ICB

T cell exclusion is a central mechanism that drives resistance to ICB. It can result from abnormal stromal structure, suppressive cancer-associated fibroblasts, and disruption in cytokine signaling pathways like WNT/β-catenin and TGF-β where cytotoxic T cells (activated/exhausted CD8^+^ T cells) are unable to infiltrate the TME (16, 30). Elevated TGF-β is associated with poor prognosis; however several preclinical studies and clinical observations in melanoma have shown that TGF-β blockade within the TME increases T cell infiltration and enhances anti-PD-1 efficacy (30, 31). When T cells fail to infiltrate the TME, there are insufficient cytotoxic T lymphocytes (CTLs) to engage the PD-1/PD-L1 axis, resulting in resistance of ICB therapy targeting PD-1 or PD-L1.

Interferons (IFNs) are key modulators of anti-tumor immunity and among the most important cytokines in cancer elimination processes. The type I/II IFN response acts through the Janus kinase (JAK)/signal transducer and activator of transcription (STAT) pathway, using these mediators to drive intracellular programs that initiate and shape the immune response (32). Specifically, type I IFN (IFN-α/β) signaling plays a critical role in anti-tumor immunity by promoting dendritic cell maturation, antigen presentation and CTL priming and recruitment (2). Impairment of type I IFN response can reduce the number of CD8^+^ T cells with PD-1 receptors, in turn diminishing or blunting the anti-PD-1 response (33). Similarly, type II IFN (IFN-γ) induces MHC I expression and upregulates PD-L1 on competent APCs (6, 34). Taube and colleagues revealed the role of type II IFN in maintaining PD-L1 expression and how elevated PD-L1 in the TME directly engages with PD-1 to cause dysfunction (35). This mechanism by which tumor cells adapt in the TME is an example of cancer immunoediting, in which the immune system both eliminates and promotes tumor growth by exerting evolutionary pressures that select for resistant clones.

In addition, accumulation of immunosuppressive cell populations such as MDSCs, TAMs, and Tregs within the TME reduces responsiveness to ICB by immune escape (36, 37). MDSCs have been known as a poor prognostic indicator and targeting them has shown to increase immunotherapy in some clinical trials (38, 39). MDSCs are also a key source of TAMs, which are the most abundant immune population in the TME and have also been implicated as a predictor of patient outcomes (40). TAMs and MDSCs secrete the inhibitory cytokines TGF-β and IL-10, which then suppress antigen presentation, inhibit effector T-cell activity, and promote T cell exhaustion, all leading to reduced response to checkpoint blockade (41). Finally, LAG-3 on T cells suppresses T cells by engaging with MHC II on Tregs, and dysfunctional T cells within the TME have higher LAG-3 expression; therefore, LAG-3 blocking antibodies are in development to restore T cell activity (42).

## Tumor-intrinsic innate immune induction via viral mimicry as a strategy to overcome ICB resistance

Viral mimicry is the phenomenon that occurs when cytoplasmic accumulcation of nucleic acid is detected, as happens when a cell is infected by virus; it induces type I IFN response and recruits immune cells to the vicinity of the cells (43, 44). Thus, viral mimicry has attracted attention as a promising approach to convert cold TME into hot ones and thereby enhance the efficacy of ICB therapy (43, 45, 46). Although many studies of viral mimicry have mainly focused on dsRNA sensing via the RIG-I/MDA5-MAVS pathway, recent work has also highlighted contributions from dsDNA (and DNA/RNA hybrid) sensing through the cyclic GMP-AMP synthase (cGAS)-stimulator of interferon genes (STING) pathway (47, 48).

Endogenous dsRNA typically accumulates when expression of repetitive elements is derepressed (49). Repetitive elements in the human genome are categorized into tandem repeats and interspersed repeats (50); the latter are frequent sources of inflammatory dsRNA (49, 51, 52). Interspersed repeats are derived from transposable elements and distributed throughout the genome. While most transposable elements have been inactivated through evolution, a portion remain transcriptionally competent. Major interspersed repeats include long interspersed nuclear elements (LINEs; ~ 6,000 bp), short interspersed nuclear elements (SINEs; ~ 300 bp), and long terminal repeats (LTRs) that often are remnants of endogenous retroviruses (ERVs) (49, 53). In normal cells, repetitive elements are transcriptionally silenced to protect genomic integrity, but their expression is frequently deregulated in cancer cells due to loss of DNA methylation or repressive histone modifications (50, 54). This deregulation can be further accelerated by therapeutically targeting epigenetic factors, as detailed in the following section.

Upon recognition of cytoplasmic dsDNA, cGAS catalyzes the formation of cyclic GMP-AMP (cGAMP) from GTP and ATP (55). Binding of cGAMP to STING activates type I IFN signaling through phosphorylation of TBK1 and IRF3 (56, 57). Sources of cGAS-stimulatory dsDNA include formed micronuclei as a consequence of DNA damage and leaked mitochondrial DNA (58, 59). Thus, genomically unstable tumors are frequently linked to activation of immunogenic signatures and hot TMEs (60–62). Recently, Crossley et al. further demonstrated that cytoplasmic DNA/RNA hybrids, generated when DNA damage is induced by R-loop accumulation, can also activate the cGAS-STING pathway (63). Maxwell and colleagues revealed that loss of ARID1A, observed in patients with favorable response to ICB, induces R-loop accumulation and STING activation via R-loop derived cytosolic DNA, further supporting a link between R-loops and the cGAS-STING pathway (64). Elevated expression of LINE L1 elements, which encode proteins required for transposition (65), can also enhance the formation of reverse-transcribed DNA/RNA hybrid intermediates, providing another route to cGAS activation (66). These results are consistent with prior evidence that cGAS can directly bind DNA/RNA hybrids and synthesize cGAMP (67), and R-loop is gathering attention as a key driver of immune response (59, 68).

**Figure 1:**
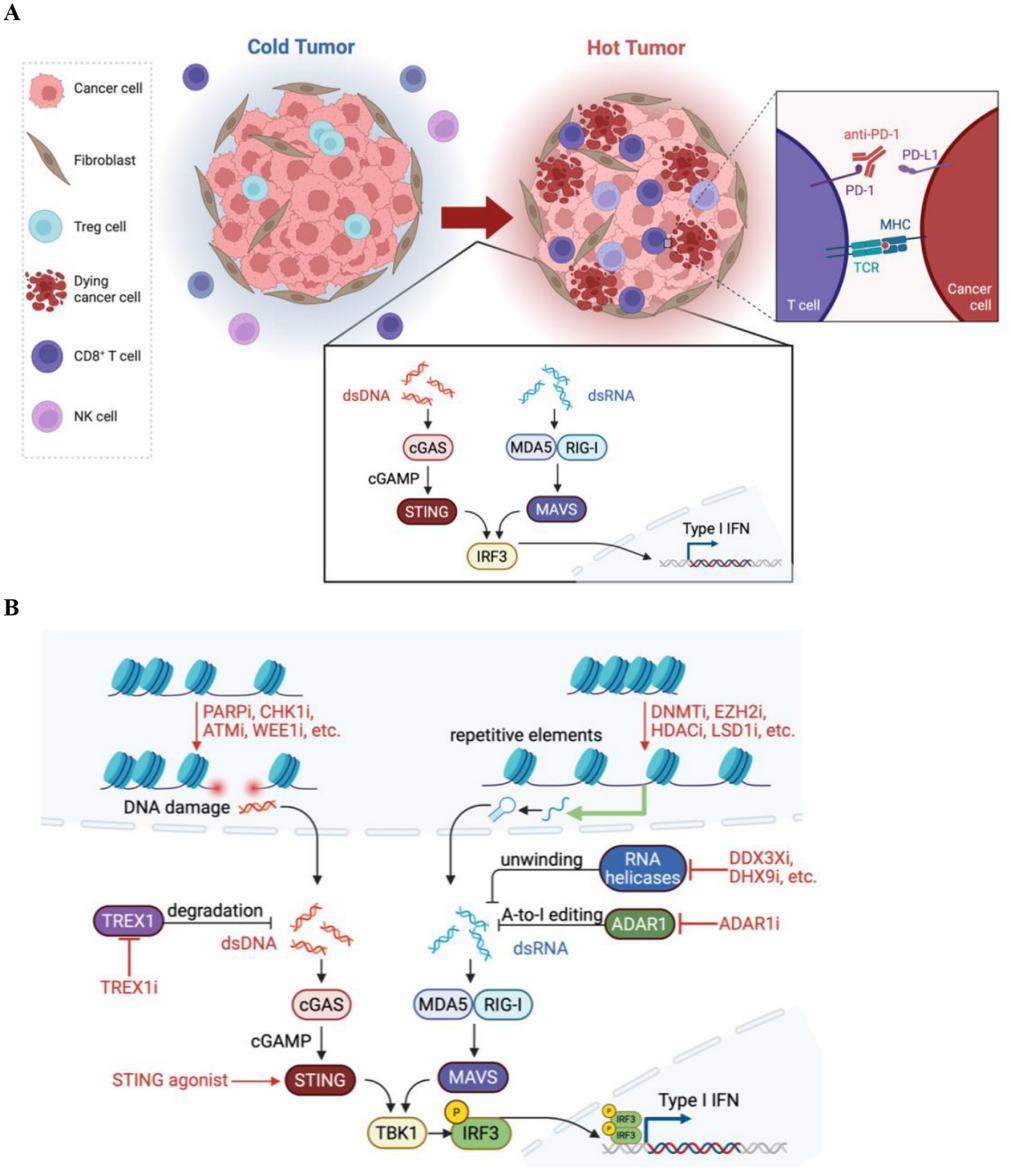
**(A)** General schematic of “cold” vs. “hot” tumor microenvironment (TME) and viral mimicry activation. “cold” tumor illustration observing limited immune cell infiltration and “hot” tumor illustration observing increased T cell availability and activation; IFN response induced by either cytoplasmic dsDNA-cGAS-STING pathway or dsRNA-RIG-I/MDA5-MAVS pathway is a promising way to make “hot” TME. **(B)** Schematic of inhibitors that can induce viral mimicry through either the cGAS-STING pathway or the MDA-RIG-I-MAVS pathway, ultimately triggering a type I IFN response. Created with BioRender.com.

Thus, viral mimicry in cancer cells can be driven by multiple mechanisms that increase cytoplasmic dsRNA, dsDNA, or DNA/RNA hybrids, engaging both RIG-I/MDA5-MAVS and cGAS-STING pathways to elicit innate immune responses (69–73). Understanding the specific regulators of these nucleic acids in cancer cells is critical for designing therapies that efficiently induce viral mimicry (Figures 1A, B).

One important point to note is that acute induction of viral mimicry is critical to convert “cold” tumors into “hot,” because chronic activation of innate immune pathways can sometimes lead to immune suppression, T cell exhaustion, or adaptive resistance. For example, sustained STING activation produces prolonged type I IFN and inflammatory cytokine release, which can foster an immunosuppressive TME and drive upregulation of immune checkpoint molecules (60, 74, 75). Thus, when therapeutically inducing viral mimicry, it is essential to carefully determine an appropriate duration of drug treatment.

Additionally, some cancers possess or develop tolerance to viral mimicry itself. Ishak and colleagues showed that loss of the tumor suppressor p53 enables cancer cells to possess tolerance to DNMTi via chronic activation of the IFN response (76). Qin et al. (77) reported that prolonged IFN signaling in cancer cells can induce immune dysfunction through epigenetic reprogramming. Thus, both intrinsic and acquired resistance mechanisms to viral mimicry warrant careful attention.

Viral mimicry and oncolytic virotherapy share overlapping mechanisms. Oncolytic virotherapy uses genetically modified viruses to induce tumor-intrinsic innate immunity through viral RNA/DNA accumulation (78–80), while viral mimicry leverages endogenous viral-like nucleic acids. These approaches can be complementary; viral mimicry may prime a “hotter” TME that enhances antigen presentation, while oncolytic virotherapy can amplify innate and adaptive responses initiated by mimicry and additionally debulk tumors (81, 82). Combining targeted viral-mimicry agents with carefully selected oncolytic platforms could maximize immune activation while allowing dose sparing and improved safety (82).

## Therapeutic approaches to induce viral mimicry

### DNA methylation and histone methylation related targets

Epigenetic therapies that induce derepression of repetitive elements are among the most promising strategies to induce viral mimicry. Chiappinelli and colleagues reported in 2015 that DNA methyltransferase inhibitors (DNMTi) upregulate immune signaling by derepressing ERV expression, which contributes to aberrant accumulation of dsRNAs (83). Histone methylation has also been targeted to disrupt repetitive element silencing: H3K9me3, regulated by the SUV39H histone lysine methyltransferase, is a major repressive mark at ERV and LINE loci (84). Genetic depletion and pharmacologic inhibition of SUV39H1 promotes CD8^+^ T cell infiltration by altering the chromatin state of cancer cells (85). Shen et al. reported that inhibition of FBXO44, which is essential for SUV39H1-dependent H3K9me3, efficiently triggers antiviral pathways in cancer cells via repetitive element transcription to overcome resistance to ICB therapy (47). Other histone methyltransferases, including EZH2 and G9a, are also promising targets to potentiate ICB therapy (86, 87). Histone deacetylase (HDAC) inhibitors (which increase global histone acetylation) and lysine-specific demethylase 1 (LSD1) inhibitors (which alter H3K4 and H3K9 methylation) have likewise been used to deregulate ERV expression (88, 89). More recently, Zhao et al. identified trinucleotide-repeat-containing 18 (TNRC18) as an H3K9me3 reader involved in ERV silencing and showed that a point mutation in TNRC18 leads to derepressed expression of ERVs (90). A deeper understanding of epigenetic regulation of repetitive elements will enable more specific and effective therapeutic approaches.

### RNA metabolism related targets

Agents that perturb nucleic acid metabolism can also induce dsRNA accumulation in cancer cells. Adenosine deaminase acting on RNA 1 (ADAR1) destabilizes and prevents accumulation of inverted repeat Alu dsRNA with its adenosine to inosine (A-to-I) editing activity (91). Thus, ADAR1 depletion triggers tumor-intrinsic innate immunity through dsRNA accumulation (92). Therapeutic ADAR1 inhibitors are in active development (93–95). RNA helicases that unwind dsRNA structures into single-strand RNA are also important regulators of dsRNA homeostasis. Genetic depletion of DDX3X or DHX9 leads to accumulation of cytoplasmic dsRNA, which activates type I IFN signaling via dsRNA-sensing pathways (96–99). We and other groups have reported that loss of DHX9 leads to R-loop accumulation, suggesting that targeting this molecule could also be a promising strategy to activate the cGAS-STING pathway (97, 99–101). In addition, Zou et al. showed that XRN1, a 5′-3′ exoribonuclease, is a negative regulator of dsRNA; deletion of XRN1 gene in cancer cells induces upregulation of IFN stimulated genes (102).

### DNA metabolism related targets

Three prime repair exonuclease 1 (TREX1) is a key repressor of the cGAS-STING pathway; by degrading cytoplasmic DNA with its exonuclease activity, it prevents accumulation of DNA. Mutations in TREX1 are strongly linked to autoimmune diseases by causing self-DNA accumulation (103, 104). Cancer cells often upregulate TREX1 in response to drug treatment to suppress tumor-intrinsic innate immune responses (105, 106). Consequently, this molecule has emerged as a promising target: its inhibition leads to cytoplasmic dsDNA accumulation and activation of the cGAS-STING pathway (105–108). To more directly stimulate the STING pathway in cancer cells, STING agonists are actively being developed, and several have shown impressive potential in preclinical studies (109, 110). Although challenges with the agonists remain – for example, the vulnerability of enzymatic degradation of the agonists and the potential risk of cytokine release syndrome caused by systemic exposure – this approach remains one of the promising strategies to improve ICB efficacy (111–113).

### DNA damage inducers

Radiotherapy and chemotherapy are often combined with ICB therapy (114, 115) because DNA damage leads to accumulation of cytoplasmic DNA and induction of tumor-intrinsic innate immunity (116). The status of the DNA damage response, such as tumor mutational burden, microsatellite instability, and deficient DNA mismatch repair, can be used as biomarkers to select patients for immunotherapy (117, 118). Targeted therapies that induce DNA damage are also a promising means to trigger tumor-intrinsic innate immune responses. Sen et al. reported that targeting the DNA damage response proteins PARP or checkpoint kinase 1 (CHK1) increases cytoplasmic DNA and enhances response to PD-L1 blockade through the cGAS-STING pathway activation in small cell lung cancers (119). Inhibitors of other DNA damage response proteins, including ATM, ATR, DNA-PK, and WEE1, are also promising combination partners to induce innate immune responses (117, 120, 121).

## Clinical translation of viral mimicry to overcome ICB resistance

Combining viral mimicry strategies with ICB therapy is now being evaluated clinically. DNMTi are approved for higher risk myelodysplastic syndrome (MDS), chronic myelomonocytic leukemia (CMML), and acute myeloid leukemia (AML) (122). Consequently, combinations of DNMTi and ICB have been tested for these diseases in early clinical trials (123). A phase II trial of the DNMT inhibitor azacitidine plus the anti-PD-1 antibody nivolumab for relapsed/refractory AML showed encouraging response rates and improved overall survival (124). Two phase II trials for MDS have been encouraging: azacitidine with the anti-PD-1 antibody pembrolizumab demonstrated that the combination is safe with manageable toxicities (125), while the DNMTi decitabine with the anti-PD-1 antibody sintilimab showed promising efficacy and tolerability (126). Guadecitabine, a next-generation DNMTi, was tested in combination with the PD-L1 inhibitor atezolizumab against relapsed/refractory MDS and CMML, showing modest efficacy and manageable adverse events (127).

Combinations of DNMTi and ICB are also being explored in solid tumors. A phase I dose-escalation study of guadecitabine plus pembrolizumab across various solid tumors found the regimen tolerable with evidence of biological and anti-tumor activity (128). In a phase II trial, guadecitabine combined with atezolizumab in metastatic urothelial carcinoma induced immune activation in a subset of patients, which correlated with longer progression-free survival (129). In classic Hodgkin's lymphoma, decitabine combined with camrelizumab was evaluated in a phase II trial, and the combination was associated with better progression-free survival (130). Azacitidine with the PD-L1 inhibitor durvalumab and CTLA-4 inhibitor tremelimumab was tested in recurrent/metastatic head and neck squamous cell carcinoma (phase Ib) and induced upregulation of immune-response gene pathways in the treated tumors, with limited adverse effects (131). Beyond DNMTi, inhibitors of EZH2 are being tested in combination with ICB (86). LSD1 inhibitors are evaluated in monotherapy and in combination with ICB (132, 133). Development of STING agonists is a major focus, with compounds currently tested in clinical trials against solid tumors or refractory tumors (134). Most STING agonists are still in phase I, and it's critical to set correct dosage because excessive activation by too much agonist can provoke systemic inflammation and autoimmunity (135). Inhibitors targeting TREX1, RNA helicases (99, 101, 136), and ADAR1 (93–95) are also under preclinical or early clinical investigation.

Viral mimicry is a promising strategy but can yield inconsistent or disappointing results. Like other immunotherapies, viral mimicry seeks to enhance immune recognition of cancer cell. Thus, compared with conventional chemotherapy or radiotherapy, which directly kill cells or inhibit proliferation, its acute cytotoxic side effects are often milder (137). However, autoimmune toxicities and systemic inflammation can occur. For example, DNMTis have been reported to promote CD4^+^ T cells autoreactivity by altering TCR recongnition of self MHC molecules (138, 139). In addition, because loss-of-function mutations in TREX1 cause systemic lupus erythematosus (SLE) and Aicardi-Goutieres syndrome (AGS), long-term inhibition of TREX is expected to carry significant immunological risks (140, 141). Overall, the central challenge of viral mimicry is finding a dose that induces anti-tumor immunity while minimizing adverse effects such as autoimmunity.

Defining biosmarkers is critical to identify patients likely to benefit and to exclude those who will have disappointing outcomes. Global DNA methylation level, which can be altered by IDH1/2 mutations, will be useful markers when using methylation-targeting agents (142, 143). For strategies targeting ADAR1, TREX1, or RNA helicases, the expression levels of these proteins are likely to be important predictive biomarkers (105). Likewise, because many cancers downregulate STING to suppress tumor-intrinsic innate immunity, assessing STING protein expression is essential when considering therapies aimed at the cGAS-STING pathway (144–146).

## Discussion

In this review, we summarize known and recent findings on how to induce an immune response in otherwise resistant tumors. Despite the advances and success in ICB, there is only a subset of patients where durable clinical responses are observed, emphasizing the importance of understanding TME-mediated resistance mechanisms. Across several tumor types, resistance is induced when anti-tumor immune activation is impaired whether that be through T-cell exclusion, impaired antigen presentation, cancer immunoediting, or accumulation of immunosuppressive cells (2, 13). These mechanisms altogether will establish a “cold” TME that limits the ability of ICB therapies to restore cytotoxic T cell responses (10, 14, 15).

Viral mimicry represents a compelling strategy to convert immunologically “cold” tumors into “hot” ones by promoting cytosolic accumulation of nucleic acids and activating RIG-I/MDA5-MAVS and cGAS-STING pathways. Preclinical and early clinical data support multiple avenues - epigenetic derepression of repetitive elements (DNMT, H3K9/EZH2/G9a, HDAC, LSD1 inhibitors), inhibition of RNA/DNA metabolism (ADAR1, XRN1, DDX helicases, TREX1), direct STING agonism, and DNA damage-inducing agents - that elicit innate immune signaling and sensitize tumors to immune checkpoint blockade (Table 1).

**Table 1:** Summary of therapeutic approaches that induce viral mimicry by cancer/tumor type, research status, biomarkers, and representative clinical trials.

Therapeutic approach	Mechanism(s) of action	Cancer/Tumor types	*Pre-clinical*	Representative therapeutics and trial phases	Suggested biomarkers	Limitations
Immune checkpoint blockade (ICB) therapy	Anti-PD antibodies bind to PD-1 on T-cells while anti-PD-L1 antibodies bind PD-L1 on tumor/immune cells to block checkpoint signaling and restore CD8^+^ effector T-cells.	Metastatic Melanoma, Hodgkin's Lymphoma, Small Cell Lung Cancer	(34–37, 136)	Phase II: Pembrolizumab and Nivolumab; Camrelizumab, and Sintilimab	TGF-β; PD-L1; MDSCs; PTEN	Primary and acquired resistance along with immune-mediated adverse effects in 10–15% of patients (2, 4, 5); Only 40% response rate with nivolumab (4); PTEN study had a small patient cohort (30).
	Stimulated naïve CD8^+^ T cells differentiate into CTLs who infiltrate and kill cancer cells, therefore anti-CTL-4 antibodies mediate this process allowing T-cell proliferation and secretion of inflammatory cytokines triggering tumor-cell death through perforin/granzyme pathways.	Metastatic Melanoma, Head and Neck Squamous Cell Carcinoma (SCC)	(34–37)	Phase I/Ib: Azacitidine + Durvalumab; Ipilimumab; Tremelimumab	CD8+; CTLA-4	Broad toxicities in normal organs/tissues; limited efficacy beyond melanoma patients (4). Limited adverse hematologic effects in head/neck SCC when combined with DNMTis (109).
	The inhibitory receptor found on T cells and NK cells LAG-3, binds to MHC-I and exhausts cells, preventing them from differentiation for attack, therefore inhibition of LAG-3 allows for T cell proliferation and attack on cancer cells	Melanoma, colorectal cancer, adenocarcinoma (*in-vivo*/pre-clinical)	(40–42)	Preclinical/Phase I: Relatlimab; Novel Anti-LAG-3 combined with nivolumab/pembrolizumab	LAG-3; MHC I/II	When combined with nivolumab, dose escalation, higher immune mediated adverse effects along with rash, fatigue, joint pain, and nausea (9, 147).
DNA methyltransferase inhibitors (DNMTis) and histone deacetylase/demethylase inhibitors	DNMTis and histone deacetylase/demethylase inhibition cause derepression of ERV expression to trigger accumulation of dsRNAs and subsequent tumor-intrinsic immunity.	Myelodysplastic syndrome (MDS), chronic myelomonocytic leukemia (CMML), Acute myeloid leukemia (AML), and Head and Neck Squamous Cell Carcinoma (SCC), Hodgkin's Lymphoma	(103–105, 107, 109)	Phase II: DNMTi + Azacitidine, and Nivolumab; Guadecitabine; Decitabine + ICB; Tazemetostat (EZH2 inhibitor); LSD1 inhibitors + ICB therapy.	EZH2; LSD1; FBXO44; IDH 1/2	Severe cytopenia adverse effects in AML and MDS; myelosuppression in solid tumors due to already compromised bone marrow function; Significant immunological risks like systemic lupus erythematosus (103–105, 107, 109, 140, 141)
RNA metabolism inhibition	Adenosine deaminase acting on RNA 1 (ADAR1) depletion/inhibition induces accumulation of inverted repeat Alu dsRNA, triggering tumor-intrinsic innate immunity; Targeting RNA helicases also induces cytoplasmic accumulation of dsRNA.	Melanoma, Glioblastoma, Breast Cancer and mouse ovarian cancer cell lines	(74–80, 91–102)	Preclinical/Phase I: PRMT5i; 8-azanebularine	ADAR1; DDX3X; DHX9; XRN1	None in animal models, thrombocytopenia in 20% of patients, and between 20–40% of patients experienced nausea/fatigue/dysgeusia, asthenia and diarrhea (148).
DNA metabolism inhibition	Induction of accumulated cytoplasmic dsDNA to activate the cGAS-STING pathway and trigger IFN response	Solid tumors, Non-small cell lung cancer, Triple Negative Breast Cancer, and Melanomas	(74, 105–108)	Phase I: STING agonists; Phase I/II: Platinum based therapy (paclitaxel, carboplatin + ICB therapy – ipilimumab, pembrolizumab, and nivolumab)	TBK1/IRF3; ARID1A; TREX1; STING	Enzymatic degradation of agonists with risk of cytokine release, SLE, AGS, varying response in overall survival and a safe delivery system (111–113, 140, 141)
DNA damage inducers	Induction of DNA damage by targeting DNA damage repair (DDR) proteins, triggering tumor-intrinsic immune response by accumulation of cytoplasmic DNA	Small Cell Lung Cancer, Ovarian tumors, Melanoma, Triple Negative Breast Cancer	(60–64, 121)	Phase I/II: Radiation, platinum-based chemotherapy (Cisplatin and Carboplatin), DNA damage response inhibitors (PARP; CHK1; WEE1) + ICB therapy	Microsatellites (short tandem repeats/simple sequence repeats); DNA damage repair proteins (PARP, CHK1, ATM, ATR, DNA-PK and WEE1)	Dose-dependent effects vary widely across tumor types; durable responses vary (117, 121).

However, therapeutic translation faces key challenges: defining biomarkers that predict which nucleic-acid source and sensing axis dominate in each tumor (e.g., ADAR1, TREX1, STING, or global methylation status), balancing anti-tumor immunity with deleterious systemic inflammation or autoimmunity, optimizing dosing and delivery (e.g., limiting systemic toxicity of STING agonists) (135), and overcoming tumor adaptation such as upregulation of immune suppressive pathways and development of tolerance to viral mimicry (60, 74, 76). Future work should integrate rigorous mechanistic studies with biomarker-driven clinical trials, explore rational combinations (e.g., targeted epigenetic or metabolic modulators plus ICB), and develop selective modulators of nucleic-acid regulators to maximize efficacy while minimizing toxicity. Such a strategy could broaden the therapeutic window for ICB across resistant cancers.

## References

[1] Pardoll DM. The blockade of immune checkpoints in cancer immunotherapy. Nat Rev Cancer. (2012) 12:252–64. doi: 10.1038/nrc3239 22437870 PMC4856023

[2] Vesely MD Zhang T Chen L. Resistance mechanisms to anti-PD cancer immunotherapy. Annu Rev Immunol. (2022) 40:45–74. doi: 10.1146/annurev-immunol-070621-030155 35471840

[3] Rabinovich GA Gabrilovich D Sotomayor EM. Immunosuppressive strategies that are mediated by tumor cells. Annu Rev Immunol. (2007) 25:267–96. doi: 10.1146/annurev.immunol.25.022106.141609 17134371 PMC2895922

[4] Hodi FS O'Day SJ McDermott DF Weber RW Sosman JA Haanen JB . Improved survival with ipilimumab in patients with metastatic melanoma. N Engl J Med. (2010) 363:711–23. doi: 10.1056/NEJMx100063 20525992 PMC3549297

[5] Robert C Long GV Brady B Dutriaux C Maio M Mortier L . Nivolumab in previously untreated melanoma without BRAF mutation. N Engl J Med. (2015) 372:320–30. doi: 10.1056/NEJMoa1412082 25399552

[6] Chen L Han X. Anti-PD-1/PD-L1 therapy of human cancer: past, present, and future. J Clin Invest. (2015) 125:3384–91. doi: 10.1172/JCI80011 26325035 PMC4588282

[7] Nishino M Ramaiya NH Hatabu H Hodi FS. Monitoring immune-checkpoint blockade: response evaluation and biomarker development. Nat Rev Clin Oncol. (2017) 14:655–68. doi: 10.1038/nrclinonc.2017.88 28653677 PMC5650537

[8] Rotte A. Combination of CTLA-4 and PD-1 blockers for treatment of cancer. J Exp Clin Cancer Res. (2019) 38:255. doi: 10.1186/s13046-019-1259-z 31196207 PMC6567914

[9] Paik J. Nivolumab plus relatlimab: first approval. Drugs. (2022) 82:925–31. doi: 10.1007/s40265-022-01723-1 35543970

[10] Marchesi S Marinello A Ambrosini P Cavalli C Lo Russo G Occhipinti M. Immune-checkpoint targeting drug conjugates: a novel class of promising therapeutic agents for cancer treatment. NPJ Precis Oncol. (2025) 9:219. doi: 10.1038/s41698-025-01011-7 40603576 PMC12223136

[11] Nambiar DK Maddineni S Langthasa J Cao H Viswanathan V Liu J . VISTA immune checkpoint blunts radiotherapy-induced antitumor immune response. Cell Rep. (2025) 44:115893. doi: 10.1016/j.celrep.2025.115893 40580480 PMC12832042

[12] Bautista J Echeverría CE Maldonado-Noboa I Adatty-Molina J Suárez Urresta S Coral-Riofrio EC . Next-generation immune checkpoints and tumor microenvironment modulation in cancer immunotherapy. J Immunol Res. (2026) 2026:e7864229. doi: 10.1155/jimr/7864229 41811823 PMC13140820

[13] Zhang J Huang D Saw PE Song E. Turning cold tumors hot: from molecular mechanisms to clinical applications. Trends Immunol. (2022) 43:523–45. doi: 10.1016/j.it.2022.04.010 35624021

[14] Wu B Zhang B Li B Wu H Jiang M. Cold and hot tumors: from molecular mechanisms to targeted therapy. Signal Transduct Target Ther. (2024) 9:274. doi: 10.1038/s41392-024-01979-x 39420203 PMC11491057

[15] Hegde PS Chen DS. Top 10 challenges in cancer immunotherapy. Immunity. (2020) 52:17–35. doi: 10.1016/j.immuni.2019.12.011 31940268

[16] Aliazis K Christofifes A Shah R Yeo YY Jiang S Charest A . The tumor microenvironment's role in the response to immune checkpoint blockade. Nat Cancer. (2025) 6:924–37. doi: 10.1038/s43018-025-00986-3 40514448 PMC12317369

[17] Fridman WH Pages F Sautes-Fridman C Galon J. The immune contexture in human tumours: impact on clinical outcome. Nat Rev Cancer. (2012) 12:298–306. doi: 10.1038/nrc3245 22419253

[18] Gajewski TF Schreiber H Fu YX. Innate and adaptive immune cells in the tumor microenvironment. Nat Immunol. (2013) 14:1014–22. doi: 10.1038/ni.2703 24048123 PMC4118725

[19] Lamplugh ZL Wellhausen N June CH Fan Y. Microenvironmental regulation of solid tumour resistance to CAR T cell therapy. Nat Rev Immunol. (2026) 26:230–48. doi: 10.1038/s41577-025-01229-3 41087553

[20] Anandappa AJ Wu CJ Ott P. A directing traffic: how to effectively drive t cells into tumors. Cancer Discov. (2020) 10:185–97. doi: 10.1158/2159-8290.CD-19-0790 31974169 PMC7007384

[21] Cha JH Chan LC Li CW Hsu JL Hung MC. Mechanisms controlling PD-L1 expression in cancer. Mol Cell. (2019) 76:359–70. doi: 10.1016/j.molcel.2019.09.030 31668929 PMC6981282

[22] Doroshow DB Bhalla S Beasley MB Sholl LM Kerr KM Gnjatic S . PD-L1 as a biomarker of response to immune-checkpoint inhibitors. Nat Rev Clin Oncol. (2021) 18:345–62. doi: 10.1038/s41571-021-00473-5 33580222

[23] Winkler J Abisoye-Ogunniyan A Metcalf KJ Werb Z. Concepts of extracellular matrix remodelling in tumour progression and metastasis. Nat Commun. (2020) 11:5120. doi: 10.1038/s41467-020-18794-x 33037194 PMC7547708

[24] Rizvi NA Hellmann MD Snyder A Kvistborg P Makarov V Havel JJ . Cancer immunology. Mutational landscape determines sensitivity to PD-1 blockade in non-small cell lung cancer. Science. (2015) 348:124–8. doi: 10.1126/science.aaa1348 25765070 PMC4993154

[25] Khodadoust MS Olsson N Wagar LE Haabeth OA Chen B Swaminathan K . Antigen presentation profiling reveals recognition of lymphoma immunoglobulin neoantigens. Nature. (2017) 543:723–7. doi: 10.1038/nature21433 28329770 PMC5808925

[26] Li Y Fang M Yu H Wang X Xue S Jiang Z . Neoantigen enriched biomimetic nanovaccine for personalized cancer immunotherapy. Nat Commun. (2025) 16:4783. doi: 10.1038/s41467-025-59977-8 40404668 PMC12098835

[27] Mahadevan NR Knelson EH Wolff JO Vajdi A Saigi M Campisi M . Intrinsic immunogenicity of small cell lung carcinoma revealed by its cellular plasticity. Cancer Discov. (2021) 11:1952–69. doi: 10.1158/2159-8290.CD-20-0913 33707236 PMC8338750

[28] Dhatchinamoorthy K Colbert JD Rock KL. Cancer immune evasion through loss of MHC class I antigen presentation. Front Immunol. (2021) 12:636568. doi: 10.3389/fimmu.2021.636568 33767702 PMC7986854

[29] Chen Z Han F Du Y Shi H Zhou W. Hypoxic microenvironment in cancer: molecular mechanisms and therapeutic interventions. Signal Transduct Target Ther. (2023) 8:70. doi: 10.1038/s41392-023-01332-8 36797231 PMC9935926

[30] Trujillo JA Luke JJ Zha Y Segal JP Ritterhouse LL Spranger S . Secondary resistance to immunotherapy associated with beta-catenin pathway activation or PTEN loss in metastatic melanoma. J Immunother Cancer. (2019) 7:295. doi: 10.1186/s40425-019-0780-0 31703593 PMC6839232

[31] Ahmadi A Najafi M Farhood B Mortezaee K. Transforming growth factor-beta signaling: tumorigenesis and targeting for cancer therapy. J Cell Physiol. (2019) 234:12173–87. doi: 10.1002/jcp.27955 30537043 PMC13484046

[32] Meraz MA White JM Sheehan KC Bach EA Rodig SJ Dighe AS . Targeted disruption of the Stat1 gene in mice reveals unexpected physiologic specificity in the JAK-STAT signaling pathway. Cell. (1996) 84:431–42. doi: 10.1016/S0092-8674(00)81288-X 8608597

[33] Razaghi A Durand-Dubief M Brusselaers N Bjornstedt M. Combining PD-1/PD-L1 blockade with type I interferon in cancer therapy. Front Immunol. (2023) 14:1249330. doi: 10.3389/fimmu.2023.1249330 37691915 PMC10484344

[34] Abiko K Matsumura N Hamanishi J Horikawa N Murakami R Yamaguchi K . IFN-gamma from lymphocytes induces PD-L1 expression and promotes progression of ovarian cancer. Br J Cancer. (2015) 112:1501–9. doi: 10.1038/bjc.2015.101 25867264 PMC4453666

[35] Taube JM Anders RA Young GD Xu H Sharma R McMiller TL . Colocalization of inflammatory response with B7-h1 expression in human melanocytic lesions supports an adaptive resistance mechanism of immune escape. Sci Transl Med. (2012) 4:127ra137. doi: 10.1126/scitranslmed.3003689 22461641 PMC3568523

[36] Arce Vargas F Furness AJS Solomon I Joshi K Mekkaoui L Lesko MH . Fc-Optimized anti-CD25 depletes tumor-infiltrating regulatory T cells and synergizes with PD-1 blockade to eradicate established tumors. Immunity. (2017) 46:577–86. doi: 10.1016/j.immuni.2017.03.013 28410988 PMC5437702

[37] Li T Liu T Zhu W Xie S Zhao Z Feng B . Targeting MDSC for immune-checkpoint blockade in cancer immunotherapy: current progress and new prospects. Clin Med Insights Oncol. (2021) 15:11795549211035540. doi: 10.1177/11795549211035540 34408525 PMC8365012

[38] Okla K Czerwonka A Wawruszak A Bobinski M Bilska M Tarkowski R . Clinical relevance and immunosuppressive pattern of circulating and infiltrating subsets of myeloid-derived suppressor cells (MDSCs) in epithelial ovarian cancer. Front Immunol. (2019) 10:691. doi: 10.3389/fimmu.2019.00691 31001284 PMC6456713

[39] Weide B Martens A Zelba H Stutz C Derhovanessian E Di Giacomo AM . Myeloid-derived suppressor cells predict survival of patients with advanced melanoma: comparison with regulatory T cells and NY-ESO-1- or melan-A-specific T cells. Clin Cancer Res. (2014) 20:1601–9. doi: 10.1158/1078-0432.CCR-13-2508 24323899

[40] Xiang X Wang J Lu D Xu X. Targeting tumor-associated macrophages to synergize tumor immunotherapy. Signal Transduct Target Ther. (2021) 6:75. doi: 10.1038/s41392-021-00484-9 33619259 PMC7900181

[41] Mantovani A Marchesi F Malesci A Laghi L Allavena P. Tumour-associated macrophages as treatment targets in oncology. Nat Rev Clin Oncol. (2017) 14:399–416. doi: 10.1038/nrclinonc.2016.217 28117416 PMC5480600

[42] Williams JB Horton BL Zheng Y Duan Y Powe JD Gajewski TF. The EGR2 targets LAG-3 and 4-1BB describe and regulate dysfunctional antigen-specific CD8+ T cells in the tumor microenvironment. J Exp Med. (2017) 214:381–400. doi: 10.1084/jem.20160485 28115575 PMC5294847

[43] Chen R Ishak CA De Carvalho DD. Endogenous retroelements and the viral mimicry response in cancer therapy and cellular homeostasis. Cancer Discov. (2021) 11:2707–25. doi: 10.1158/2159-8290.CD-21-0506 34649957

[44] Sun S. Greenbaum BD. Viral mimicry by repeats mediates evolutionary trade-offs in cancer-immune co-evolution. Cancer Discov. (2026). doi: 10.1158/2159-8290.CD-25-2187. [Epub ahead of print]. 41997582

[45] Rosenberg L Vabret N. Viral mimicry in cancer therapy. Trends Cancer. (2025) 11:1185–202. doi: 10.1016/j.trecan.2025.08.010 40987678

[46] Mehdipour P Marhon SA Ettayebi I Chakravarthy A Hosseini A Wang Y . Epigenetic therapy induces transcription of inverted SINEs and ADAR1 dependency. Nature. (2020) 588:169–73. doi: 10.1038/s41586-020-2844-1 33087935

[47] Shen JZ Qiu Z Wu Q Finlay D Garcia G Sun D . FBXO44 promotes DNA replication-coupled repetitive element silencing in cancer cells. Cell. (2021) 184:352–69 e323. doi: 10.1016/j.cell.2020.11.042 33357448 PMC8043252

[48] Feng S Marhon SA Sokolowski DJ D'Costa A Soares F Mehdipour P . Inhibiting EZH2 targets atypical teratoid rhabdoid tumor by triggering viral mimicry via both RNA and DNA sensing pathways. Nat Commun. (2024) 15:9321. doi: 10.1038/s41467-024-53515-8 39472584 PMC11522499

[49] Sadeq S Al-Hashimi S Cusack CM Werner A. Endogenous Double-Stranded RNA. Noncoding RNA. (2021) 7:15. doi: 10.3390/ncrna7010015 33669629 PMC7930956

[50] Liao X Zhu W Zhou J Li H Xu X Zhang B . Repetitive DNA sequence detection and its role in the human genome. Commun Biol. (2023) 6:954. doi: 10.1038/s42003-023-05322-y 37726397 PMC10509279

[51] Liu X Che L Weng H. Endogenous double-stranded RNA: bridging immune activation and cancer therapeutics. Cell Investig. (2025) 1:100018. doi: 10.1016/j.clnves.2025.100018

[52] Deblois G Tonekaboni SAM Grillo G Martinez C Kao Y Tai F . Epigenetic switch-induced viral mimicry evasion in chemotherapy-resistant breast cancer. Cancer Discov. (2020) 10:1312–29. doi: 10.1158/2159-8290.CD-19-1493 32546577

[53] Wu F Danzeng Q Wu R Shen Y Shi G. Endogenous retroviruses and response to immune checkpoint inhibitors: mechanisms, clinical evidence, and therapeutic implications. Front Immunol. (2026) 17:1835325. doi: 10.3389/fimmu.2026.1835325 42245637 PMC13230102

[54] Criscione SW Zhang Y Thompson W Sedivy JM Neretti N. Transcriptional landscape of repetitive elements in normal and cancer human cells. BMC Genomics. (2014) 15:583. doi: 10.1186/1471-2164-15-583 25012247 PMC4122776

[55] Sun L Wu J Du F Chen X Chen Z. J Cyclic GMP-AMP synthase is a cytosolic DNA sensor that activates the type I interferon pathway. Science. (2013) 339:786–91. doi: 10.1126/science.1232458 23258413 PMC3863629

[56] Barbie DA Tamayo P Boehm JS Kim SY Moody SE Dunn IF . Systematic RNA interference reveals that oncogenic KRAS-driven cancers require TBK1. Nature. (2009) 462:108–12. doi: 10.1038/nature08460 19847166 PMC2783335

[57] Tanaka Y Chen ZJ. STING specifies IRF3 phosphorylation by TBK1 in the cytosolic DNA signaling pathway. Sci Signal. (2012) 5:ra20. doi: 10.1126/scisignal.2002521 22394562 PMC3549669

[58] Kwon J Bakhoum SF. The cytosolic DNA-sensing cGAS-STING pathway in cancer. Cancer Discov. (2020) 10:26–39. doi: 10.1158/2159-8290.CD-19-0761 31852718 PMC7151642

[59] Guo J Lu M Wang C Wang D Ma T. Nucleic acid diversity in cGAS-STING pathway activation and immune dysregulation. Biomedicines. (2025) 13:2158. doi: 10.3390/biomedicines13092158 41007720 PMC12467401

[60] Hong C Schubert M Tijhuis AE Requesens M Roorda M van den Brink A . cGAS-STING drives the IL-6-dependent survival of chromosomally instable cancers. Nature. (2022) 607:366–73. doi: 10.1038/s41586-022-04847-2 35705809

[61] Yan H Lu W Wang F. The cGAS-STING pathway: a therapeutic target in chromosomally unstable cancers. Signal Transduct Target Ther. (2023) 8:45. doi: 10.1038/s41392-023-01328-4 36717545 PMC9886966

[62] Beernaert B Jady-Clark RL Shah P Ramon-Gil E Lawson NM Brodtman ZD . Chromosomal instability shapes the tumor microenvironment of esophageal adenocarcinoma via a cGAS-chemokine-myeloid axis. Sci Adv. (2026) 12:eaeb1611. doi: 10.1126/sciadv.aeb1611 41811963 PMC12978254

[63] Crossley MP Song C Bocek MJ Choi J-H Kousouros JN Sathirachinda A . R-loop-derived cytoplasmic RNA-DNA hybrids activate an immune response. Nature. (2023) 613:187–94. doi: 10.1038/s41586-022-05545-9 36544021 PMC9949885

[64] Maxwell MB Hom-Tedla MS Yi J Li S Rivera SA Yu J . ARID1A suppresses R-loop-mediated STING-type I interferon pathway activation of anti-tumor immunity. Cell. (2024) 187:3390-3408.e19. 38754421 10.1016/j.cell.2024.04.025 PMC11193641

[65] Feng Q Moran JV Kazazian HH Jr Boeke JD. Human L1 retrotransposon encodes a conserved endonuclease required for retrotransposition. Cell. (1996) 87:905–16. doi: 10.1016/S0092-8674(00)81997-2 8945517

[66] Mehmood A Xue YZ Yassin HM Zubair M Rahmat SA Ahmad A. LINE-1 retrotransposon activation drives age-associated inflammation via cytoplasmic cDNA-STING/type I interferon signalling: therapeutic potential of reverse transcriptase inhibition. Contemp Oncol. (2025) 29:240–6. doi: 10.5114/wo.2025.152369 41098867 PMC12518201

[67] Mankan AK Schmidt T Chauhan D Goldeck M Honing K Gaidt M . Cytosolic RNA:DNA hybrids activate the cGAS-STING axis. EMBO J. (2014) 33:2937–46. doi: 10.15252/embj.201488726 25425575 PMC4282641

[68] Lee SY Kwak MJ Kim JJ. R-loops: a key driver of inflammatory responses in cancer. Exp Mol Med. (2025) 57:1455–66. doi: 10.1038/s12276-025-01495-0 40629041 PMC12322051

[69] Xu J Schwarz SD Gunasekera K Steinacher R Kusnierczyk A Hottiger MO . PARP1 inhibition in naive mouse embryonic stem cells induces viral mimicry. Nucleic Acids Res. (2026) 54:gkag537. doi: 10.1093/nar/gkag537 42234580 PMC13232494

[70] Zhang FL Yang SY Zhang YL Zhao Q Yang WX Andriani L . Targeting MORC2 activates transposable element-mediated viral mimicry and potentiates immune checkpoint blockade in triple-negative breast cancer. Mol Cancer. (2026). doi: 10.1186/s12943-026-02679-6. [Epub ahead of print]. 42210236 PMC13420440

[71] Wang Y Daddi AA Hosseini A Kato K Khalili E Lindholm HT . m(6)A modification suppresses innate anti-tumour immunity in colorectal cancer by limiting alu-derived dsRNA accumulation. Nat Commun. (2026). doi: 10.1038/s41467-026-73211-z. [Epub ahead of print]. 42135281 PMC13377058

[72] Adan-Villaescusa E Castello-Uribe B Uriarte I Santamaria E Barbero R Belzunce M . Histone methyl-transferase G9a inhibition boosts the efficacy of immune checkpoint inhibitors in experimental hepatocellular carcinoma. Cell Rep Med. (2026) 7:102717. doi: 10.1016/j.xcrm.2026.102717 41923620 PMC13130624

[73] Li Q Zhang Z Wang Y Peng X Fang Y Zhang Y . ARID1A mediates SWI/SNF-independent maintenance of heterochromatin architecture to restrain viral mimicry and immunogenicity in colon cancer. Cancer Res. (2026) 86:2344–59. doi: 10.1158/0008-5472.CAN-25-3231 41661654

[74] Benci JL Johnson LR Choa R Xu Y Qiu J Zhou Z . Opposing functions of interferon coordinate adaptive and innate immune responses to cancer immune checkpoint blockade. Cell. (2019) 178:933–48 e914. doi: 10.1016/j.cell.2019.07.019 31398344 PMC6830508

[75] Boukhaled GM Harding S Brooks DG. Opposing roles of type I interferons in cancer immunity. Annu Rev Pathol. (2021) 16:167–98. doi: 10.1146/annurev-pathol-031920-093932 33264572 PMC8063563

[76] Ishak CA Marhon SA Tchrakian N Hodgson A Yau HL Gonzaga IM . Chronic viral mimicry induction following p53 loss promotes immune evasion. Cancer Discov. (2025) 15:793–817. doi: 10.1158/2159-8290.CD-24-0094 39776167 PMC12776606

[77] Qiu J Xu B Ye D Ren D Wang S Benci JL . Cancer cells resistant to immune checkpoint blockade acquire interferon-associated epigenetic memory to sustain T cell dysfunction. Nat Cancer. (2023) 4:43–61. doi: 10.1038/s43018-022-00490-y 36646856

[78] Lin D Shen Y Liang T. Oncolytic virotherapy: basic principles, recent advances and future directions. Signal Transduct Target Ther. (2023) 8:156. doi: 10.1038/s41392-023-01407-6 37041165 PMC10090134

[79] Ribas A Dummer R Puzanov I VanderWalde A Andtbacka RHI Michielin O . Oncolytic virotherapy promotes intratumoral T cell infiltration and improves anti-PD-1 immunotherapy. Cell. (2017) 170:1109–19 e1110. doi: 10.1016/j.cell.2017.08.027 28886381 PMC8034392

[80] Forbes NE Abdelbary H Lupien M Bell JC Diallo JS. Exploiting tumor epigenetics to improve oncolytic virotherapy. Front Genet. (2013) 4:184. doi: 10.3389/fgene.2013.00184 24062768 PMC3778850

[81] Chiocca EA Rabkin SD. Oncolytic viruses and their application to cancer immunotherapy. Cancer Immunol Res. (2014) 2:295–300. doi: 10.1158/2326-6066.CIR-14-0015 24764576 PMC4303349

[82] Xiao D Zhang H Liu Y Li Y Li G Ning Y. Oncolytic viruses: advanced strategies in cancer therapy. Signal Transduct Target Ther. (2026) 11:45. doi: 10.1038/s41392-025-02343-3 41639062 PMC12873401

[83] Chiappinelli KB Strissel PL Desrichard A Li H Henke C Akman B . Inhibiting DNA methylation causes an interferon response in cancer via dsRNA including endogenous retroviruses. Cell. (2015) 162:974–86. doi: 10.1016/j.cell.2015.07.011 26317466 PMC4556003

[84] Bulut-Karslioglu A De La Rosa-Velázquez IA Ramirez F Barenboim M Onishi-Seebacher M Arand J . Suv39h-dependent H3K9me3 marks intact retrotransposons and silences LINE elements in mouse embryonic stem cells. Mol Cell. (2014) 55:277–90. doi: 10.1016/j.molcel.2014.05.029 24981170

[85] Niborski LL Guegen P Ye M Thiolat A Ramos RN Caudana P . CD8+T cell responsiveness to anti-PD-1 is epigenetically regulated by Suv39h1 in melanomas. Nat Commun. (2022) 13:3739. doi: 10.1038/s41467-022-31504-z 35768432 PMC9243005

[86] Kim HJ Cantor H Cosmopoulos K. Overcoming immune checkpoint blockade resistance via EZH2 inhibition. Trends Immunol. (2020) 41:948–63. doi: 10.1016/j.it.2020.08.010 32976740

[87] Ni Y Shi M Liu L Lin D Zeng H Ong C . G9a in cancer: mechanisms, therapeutic advancements, and clinical implications. Cancers. (2024) 16:2175. doi: 10.3390/cancers16122175 38927881 PMC11201431

[88] Goyal A Bauer J Hey J Papageorgiou DN Stepanova E Daskalakis M . DNMT and HDAC inhibition induces immunogenic neoantigens from human endogenous retroviral element-derived transcripts. Nat Commun. (2023) 14:6731. doi: 10.1038/s41467-023-42417-w 37872136 PMC10593957

[89] Sheng W Lafleur MW Nguyen TH Chen S Chakravarthy A Conway JR . LSD1 ablation stimulates anti-tumor immunity and enables checkpoint blockade. Cell. (2018) 174:549–63 e519. doi: 10.1016/j.cell.2018.05.052 29937226 PMC6063761

[90] Zhao S Lu J Pan B Fan H Byrum SD Xu C . TNRC18 engages H3K9me3 to mediate silencing of endogenous retrotransposons. Nature. (2023) 623:633–42. doi: 10.1038/s41586-023-06688-z 37938770 PMC11000523

[91] Levanon EY Eisenberg E Yelin R Nemzer S Hallegger M Shemesh R . Systematic identification of abundant A-to-I editing sites in the human transcriptome. Nat Biotechnol. (2004) 22:1001–5. doi: 10.1038/nbt996 15258596

[92] Ishizuka JJ Manguso RT Cheruiyot CK Bi K Panda A Iracheta-Vellve A . Loss of ADAR1 in tumours overcomes resistance to immune checkpoint blockade. Nature. (2019) 565:43–8. doi: 10.1038/s41586-018-0768-9 30559380 PMC7241251

[93] Mendoza HG Matos VJ Park S Pham KM Beal PA. Selective inhibition of ADAR1 using 8-azanebularine-modified RNA duplexes. Biochemistry. (2023) 62:1376–87. doi: 10.1021/acs.biochem.2c00686 36972568 PMC10804918

[94] Hong X Wei Z He L Bu Q Wu G Chen G . High-throughput virtual screening to identify potential small molecule inhibitors of the Zalpha domain of the adenosine deaminases acting on RNA 1(ADAR1). Eur J Pharm Sci. (2024) 193:106672. doi: 10.1016/j.ejps.2023.106672 38103658

[95] Minakuchi M Zhang H Cassel J Shiromoto Y Villanueva J Skordalakes E . Identification of ADAR1i-124: The first effective A-to-I RNA editing inhibitor with promising cancer therapeutic potential. iScience. (2026) 29:114615. doi: 10.1016/j.isci.2025.114615 41608661 PMC12834841

[96] Choi H Kwon J Cho MS Sun Y Zheng X Wang J . Targeting DDX3X triggers antitumor immunity via a dsRNA-mediated tumor-intrinsic type I interferon response. Cancer Res. (2021) 81:3607–20. doi: 10.1158/0008-5472.CAN-20-3790 33941613 PMC8597981

[97] Murayama T Nakayama J Jiang X Miyata K Morris AD Cai KQ . Targeting DHX9 Triggers tumor-intrinsic interferon response and replication stress in small cell lung cancer. Cancer Discov. (2024) 14:468–91. doi: 10.1158/2159-8290.CD-23-0486 38189443 PMC10905673

[98] Cottrell KA Ryu S Pierce JR Soto Torres L Bohlin HE Schab AM . Induction of viral mimicry upon loss of DHX9 and ADAR1 in breast cancer cells. Cancer Res Commun. (2024) 4:986–1003. doi: 10.1158/2767-9764.CRC-23-0488 38530197 PMC10993856

[99] Castro J Daniels MH Brennan D Johnston B Gotur D Lee Y-T . A potent, selective, small-molecule inhibitor of DHX9 abrogates proliferation of microsatellite instable cancers with deficient mismatch repair. Cancer Res. (2024) 85:758–76. doi: 10.1158/0008-5472.c.7676181 39589774 PMC11831107

[100] Liu MY Lin KR Chien YL Yang BZ Tsui LY Chu HC . ATR phosphorylates DHX9 at serine 321 to suppress R-loop accumulation upon genotoxic stress. Nucleic Acids Res. (2024) 52:204–22. doi: 10.1093/nar/gkad973 37930853 PMC10783509

[101] Herrera K Takasaki K. Murayama T. Targeting R-loops: diverse RNA helicases in R-loop resolution and their potential as targets for cancer therapy. Front Cell Dev Biol. (2026) 14:1822277. doi: 10.3389/fcell.2026.1822277 42093723 PMC13139199

[102] Zou T Zhou M Gupta A Zhuang P Fishbein AR Wei HY . XRN1 deletion induces PKR-dependent cell lethality in interferon-activated cancer cells. Cell Rep. (2024) 43:113600. doi: 10.1016/j.celrep.2023.113600 38261514 PMC10989277

[103] Stetson DB Ko JS Heidmann T. Medzhitov R. Trex1 prevents cell-intrinsic initiation of autoimmunity. Cell. (2008) 134:587–98. doi: 10.1016/j.cell.2008.06.032 18724932 PMC2626626

[104] Lee-Kirsch MA Gong M Chowdhury D Senenko L Engel K Lee Y-A . Mutations in the gene encoding the3′5′ DNA exonuclease TREX1 are associated with systemic lupus erythematosus. Nat Genet. (2007) 39:1065–7. doi: 10.1038/ng2091 17660818

[105] Tani T Mathsyaraja H Campisi M Li Z-H Haratani K Fahey CG . TREX1 inactivation unleashes cancer cell STING-interferon signaling and promotes antitumor immunity. Cancer Discov. (2024) 14:752–65. doi: 10.1158/2159-8290.CD-23-0700 38227896 PMC11062818

[106] Murayama T Mahadevan NR Meador CB Ivanova EV Pan Y Knelsn EH . Targeting TREX1 Induces innate immune response in drug-resistant small-cell lung cancer. Cancer Res Commun. (2024) 4:2399–414. doi: 10.1158/2767-9764.CRC-24-0360 39177280 PMC11391691

[107] Toufektchan E Dananberg A Striepen J Hickling JH Shim A Che Y . Intratumoral TREX1 induction promotes immune evasion by limiting type I interferon. Cancer Immunol Res. (2024) 12:673–86. doi: 10.1158/2326-6066.c.7267943.v1 38408184 PMC11148545

[108] Lim J Rodriguez R Williams K Silva J Gutierrez AG Tyler P . The exonuclease TREX1 constitutes an innate immune checkpoint limiting cGAS/STING-mediated antitumor immunity. Cancer Immunol Res. (2024) 12:663–72. doi: 10.1158/2326-6066.c.7267949.v1 38489753 PMC11148535

[109] Corrales L Glickman LH McWhirter SM Kanne DB Sivick KE Katibah GE . Direct activation of STING in the tumor microenvironment leads to potent and systemic tumor regression and immunity. Cell Rep. (2015) 11:1018–30. doi: 10.1016/j.celrep.2015.04.031 25959818 PMC4440852

[110] Najem H Lea ST Tripathi S Hurley L Che C-H William I . STING agonist 8803 reprograms the immune microenvironment and increases survival in preclinical models of glioblastoma. J Clin Invest. (2024) 134:e175033. doi: 10.1172/JCI175033 38941297 PMC11178548

[111] Melero I Castanon E Alvarez M Champiat S Marabelle A. Intratumoural administration and tumour tissue targeting of cancer immunotherapies. Nat Rev Clin Oncol. (2021) 18:558–76. doi: 10.1038/s41571-021-00507-y 34006998 PMC8130796

[112] Shi J Zhang Y Zhao N Seki E Ma L Kocic G . Precision targeting of STING: Challenges, innovations, and clinical outlook for cancer therapy. Innovation. (2026) 7:101074. doi: 10.1016/j.xinn.2025.101074 41737316 PMC12925938

[113] Wang Y Luo J Alu A Han X Wei Y Wei X. cGAS-STING pathway in cancer biotherapy. Mol Cancer. (2020) 19:136. doi: 10.1186/s12943-020-01247-w 32887628 PMC7472700

[114] Shiravand Y Khodadadi F Kashani SMA Hosseini-Fard SR Hosseini S Sadeghirad H . Immune checkpoint inhibitors in cancer therapy. Curr Oncol. (2022) 29:3044–60. doi: 10.3390/curroncol29050247 35621637 PMC9139602

[115] Lynch C Pitroda SP Weichselbaum RR. Radiotherapy, immunity, and immune checkpoint inhibitors. Lancet Oncol. (2024) 25:e352–62. doi: 10.1016/S1470-2045(24)00075-5 39089313

[116] Li T Chen ZJ. The cGAS-cGAMP-STING pathway connects DNA damage to inflammation, senescence, and cancer. J Exp Med. (2018) 215:1287–99. doi: 10.1084/jem.20180139 29622565 PMC5940270

[117] Mouw KW Goldberg MS Konstantinopoulos PA D'Andrea AD. DNA damage and repair biomarkers of immunotherapy response. Cancer Discov. (2017) 7:675–93. doi: 10.1158/2159-8290.CD-17-0226 28630051 PMC5659200

[118] Lu C Guan J Lu S Jin Q Rousseau B Lu T . DNA sensing in mismatch repair-deficient tumor cells is essential for anti-tumor immunity. Cancer Cell. (2020) 39:96–108.e6. doi: 10.1016/j.ccell.2020.11.006 33338425 PMC9477183

[119] Sen T Rodriguez BL Chen L Corte CMD Morikawa N Fujimto J . Targeting DNA damage response promotes antitumor immunity through STING-mediated T-cell activation in small cell lung cancer. Cancer Discov. (2019) 9:646–61. doi: 10.1158/2159-8290.CD-18-1020 30777870 PMC6563834

[120] Lord CJ Ashworth A. The DNA damage response and cancer therapy. Nature. (2012) 481:287–94. doi: 10.1038/nature10760 22258607

[121] Taniguchi H Caeser R Chavan SS Zhan YA Chow A Manoj P . WEE1 inhibition enhances the antitumor immune response to PD-L1 blockade by the concomitant activation of STING and STAT1 pathways in SCLC. Cell Rep. (2022) 39:110814. doi: 10.1016/j.celrep.2022.110814 35584676 PMC9449677

[122] Brunner AM Fell G Steensma D. P Historical expectations with DNA methyltransferase inhibitor monotherapy in MDS: when is combination therapy truly “promising”? Blood Adv. (2022) 6:2854–66. doi: 10.1182/bloodadvances.2021006357 35143613 PMC9092413

[123] Garcia-Manero G Gaddh M Platzbecker U Lindsley RC Larson SM Chevassut T . A phase 1 study of durvalumab as monotherapy or combined with tremelimumab with or without azacitidine in patients with myelodysplastic syndrome. Ann Hematol. (2025) 104:1577–85. doi: 10.1007/s00277-024-06081-4 40153010 PMC12031784

[124] Daver N Garcia-Manero G Basu S Boddu PC Alfayez M Cortes JE . Efficacy, safety, and biomarkers of response to azacitidine and nivolumab in relapsed/refractory acute myeloid leukemia: a nonrandomized, open-label, phase II study. Cancer Discov. (2019) 9:370–83. doi: 10.1158/2159-8290.CD-18-0774 30409776 PMC6397669

[125] Chien KS Kim K Nogueras-Gonzalez GM Borthakur G Naqvi K Daver NG . Phase II study of azacitidine with pembrolizumab in patients with intermediate-1 or higher-risk myelodysplastic syndrome. Br J Haematol. (2021) 195:378–87. doi: 10.1111/bjh.17689 34340254

[126] Wang J Li S Jiang H Chang Y-J Zhao X Jia J . Sintilimab plus decitabine for higher-risk treatment-naive myelodysplastic syndromes: efficacy, safety, and biomarker analysis of a phase II, single-arm trial. J Immunother Cancer. (2024) 12:e010355. doi: 10.1136/jitc-2024-010355 39577869 PMC11590843

[127] O'Connell CL Baer MR Orskov AD Saini SK Duong VH Kropf P . Safety, outcomes, and T-cell characteristics in patients with relapsed or refractory MDS or CMML treated with atezolizumab in combination with guadecitabine. Clin Cancer Res. (2022) 28:5306–16. doi: 10.1158/1078-0432.CCR-22-1810 36222848 PMC9772102

[128] Papadatos-Pastos D Yuan W Pal A Crespo M Ferreira A Gurel B . Phase 1, dose-escalation study of guadecitabine (SGI-110) in combination with pembrolizumab in patients with solid tumors. J Immunother Cancer. (2022) 10:e004495. doi: 10.1136/jitc-2022-004495 35717027 PMC9240883

[129] Jang HJ Hostetter G Macfarlane Madaj Z Ross EA Hinoue T . A phase II trial of guadecitabine plus atezolizumab in metastatic urothelial carcinoma progressing after initial immune checkpoint inhibitor therapy. Clin Cancer Res. (2023) 29:2052–65. doi: 10.1158/1078-0432.CCR-22-3642 36928921 PMC10233355

[130] Liu Y Wang C Li X Dong L Yang Q Chen M . Improved clinical outcome in a randomized phase II study of anti-PD-1 camrelizumab plus decitabine in relapsed/refractory Hodgkin lymphoma. J Immunother Cancer. (2021) 9:e002347. doi: 10.1136/jitc-2021-002347 33820822 PMC8025784

[131] Qin T Mattox AK Campbell JS Park JC Shi K-Y Li S . Epigenetic therapy sensitizes anti-PD-1 refractory head and neck cancers to immunotherapy rechallenge. J Clin Invest. (2025) 135:e181671. doi: 10.1172/JCI181671 40091844 PMC11910227

[132] Noce B Di Bello E Fioravanti R Mai A. LSD1 inhibitors for cancer treatment: focus on multi-target agents and compounds in clinical trials. Front Pharmacol. (2023) 14:1120911. doi: 10.3389/fphar.2023.1120911 36817147 PMC9932783

[133] Gounder M Schoffski P Jones RL Stacchiotti S Demetri GD Cote GM . Tazemetostat, an EZH2 inhibitor, in solid tumors harboring SWI/SNF alterations: a phase II basket study. Nat Commun. (2026). doi: 10.1038/s41467-026-69708-2 41882006 PMC13396634

[134] Wang B Yu W Jiang H Meng X Tang D Liu D . Clinical applications of STING agonists in cancer immunotherapy: current progress and future prospects. Front Immunol. (2024) 15:1485546. doi: 10.3389/fimmu.2024.1485546 39421752 PMC11483357

[135] Le Naour J Zitvogel L Galluzzi L Vacchelli E Kroemer G. Trial watch: STING agonists in cancer therapy. Oncoimmunology. (2020) 9:1777624. doi: 10.1080/2162402X.2020.1777624 32934881 PMC7466854

[136] Capasso A Bagby SM Dailey KL Currimjee N Yacob BW Ionkina A . First-in-class phosphorylated-p68 inhibitor RX-5902 Inhibits beta-catenin signaling and demonstrates antitumor activity in triple-negative breast cancer. Mol Cancer Ther. (2019) 18:1916–25. doi: 10.1158/1535-7163.MCT-18-1334 31488700 PMC6825586

[137] Qin S Xie B Wang Q Yang R Sun J Hu C . New insights into immune cells in cancer immunotherapy: from epigenetic modification, metabolic modulation to cell communication. MedComm. (2024) 5:e551. doi: 10.1002/mco2.551 38783893 PMC11112485

[138] Richardson B. Effect of an inhibitor of DNA methylation on T cells. II 5-Azacytidine induces self-reactivity in antigen-specific T4+ cells. Hum Immunol. (1986) 17:456–70. doi: 10.1016/0198-8859(86)90304-6 2432050

[139] Wulfing C Sumen C Sjaastad MD Wu LC Dustin ML Davis MM . Costimulation and endogenous MHC ligands contribute to T cell recognition. Nat Immunol. (2002) 3:42–7. doi: 10.1038/ni741 11731799

[140] Crow YJ Chase DS Lowenstein Schmidt J Szynkiewicz M Forte GM Gornall HL . Characterization of human disease phenotypes associated with mutations in TREX1, RNASEH2A, RNASEH2B, RNASEH2C, SAMHD1, ADAR, and IFIH1. Am J Med Genet A. (2015) 167A:296–312. doi: 10.1055/s-0036-1592307 25604658 PMC4382202

[141] Fang L Ying S Xu X Wu D. TREX1 cytosolic DNA degradation correlates with autoimmune disease and cancer immunity. Clin Exp Immunol. (2023) 211:193–207. doi: 10.1093/cei/uxad017 36745566 PMC10038326

[142] Figueroa ME Abdel-Wahab O Lu C Ward PS Patel J Shih A . Leukemic IDH1 and IDH2 mutations result in a hypermethylation phenotype, disrupt TET2 function, and impair hematopoietic differentiation. Cancer Cell. (2010) 18:553–67. doi: 10.1016/j.ccr.2010.11.015 21130701 PMC4105845

[143] Zhang Z Wang G Li Y Lei D Xiang J Ouyang L . Recent progress in DNA methyltransferase inhibitors as anticancer agents. Front Pharmacol. (2022) 13:1072651. doi: 10.3389/fphar.2022.1072651 37077808 PMC10107375

[144] Kitajima S Ivanova E Guo S Yoshida R Campisi M Sundararaman SK . Suppression of STING associated with LKB1 loss in KRAS-driven lung cancer. Cancer Discov. (2019) 9:34–45. doi: 10.1158/2159-8290.CD-18-0689 30297358 PMC6328329

[145] Zheng H Wu L Xiao Q Meng X Hafiz A Yan Q . Epigenetically suppressed tumor cell intrinsic STING promotes tumor immune escape. Biomed Pharmacother. (2023) 157:114033. doi: 10.1016/j.biopha.2022.114033 36436495 PMC9826630

[146] Sun P Han F Li X Wu C Deng T He J . Silencing PCSK9 reshapes the spatiotemporal activation of STING for safe and effective cancer immunotherapy. Nat Commun. (2025) 16:11622. doi: 10.1038/s41467-025-66630-x 41290746 PMC12749358

[147] Chocarro L Blanco E Arasanz H Fernandez-Rubio L Bocanegra A Echaide M . Clinical landscape of LAG-3-targeted therapy. Immunooncol Technol. (2022) 14:100079. doi: 10.1016/j.iotech.2022.100079 35755891 PMC9216443

[148] Feustel K Falchook GS. Protein arginine methyltransferase 5 (PRMT5) inhibitors in oncology clinical trials: a review. J Immunother Precis Oncol. (2022) 5:58–67. doi: 10.36401/JIPO-22-1 36034581 PMC9390703

